# Potential to perpetuate social biases in health care by Chinese large language models: a model evaluation study

**DOI:** 10.1186/s12939-025-02581-5

**Published:** 2025-07-15

**Authors:** Chenxi Liu, Jianing Zheng, Yushu Liu, Xi Wang, Yuting Zhang, Qiang Fu, Wenwen Yu, Ting Yu, Wang Jiang, Dan Wang, Chaojie Liu

**Affiliations:** 1 School of Medicine and Health Management, Tongji Medical College, Huazhong University of Science and Technology, 13 Hangkong Road, Wuhan, Hubei 430030 China; 2 Melbourne Institute of Applied Economic and Social Research, Faculty of Business and Economics, The University of Melbourne, 111 Barry St, Carlton, Victoria 3010 Australia; 3 Department of Community Health, School of Arts and Sciences, Tufts University, 574 Boston Ave, Suite 208, Medford, 02155 USA; 4 School of Artificial Intelligence and Automation, Huazhong University of Science and Technology, 1037 Luoyu Road, Wuhan, 430074 China; 5 Operating Room Department, Sir Run Run Shaw Hospital, Zhejiang University School of Medicine, 3 East Qingchun Road, Hangzhou, 310016 China; 6 School of Management, Hubei University of Chinese Medicine, Huangjiahu West Road No.16, Hongshan District, Wuhan, 430070 China; 7 Hubei Shizhen Laboratory, Research Center for Traditional Chinese Medicine Development, School of Management, Hubei University of Chinese Medicine, Huangjiahu West Road No.16, Hongshan District, Wuhan, 430070 China; 8 Department of Public Health, School of Psychology and Public Health, La Trobe University, 1 Kingsbury Dr, Melbourne, VIC 3086 Australia

**Keywords:** Social Bias, Large Language Models, Medical Education, Diagnosis and treatment, Patient Assessment

## Abstract

**Background:**

Large language models (LLMs) may perpetuate or amplify social biases toward patients. We systematically assessed potential biases of three popular Chinese LLMs in clinical application scenarios.

**Methods:**

We tested whether Qwen, Erine, and Baichuan encode social biases for patients of different sex, ethnicity, educational attainment, income level, and health insurance status. First, we prompted LLMs to generate clinical cases for medical education (*n* = 8,289) and compared the distribution of patient characteristics in LLM-generated cases with national distributions in China. Second, New England Journal of Medicine Healer clinical vignettes were used to prompt LLMs to generate differential diagnoses and treatment plans (*n* = 45,600), with variations analyzed based on sociodemographic characteristics. Third, we prompted LLMs to assess patient needs (*n* = 51,039) based on clinical cases, revealing any implicit biases toward patients with different characteristics.

**Results:**

The three LLMs showed social biases toward patients with different characteristics to varying degrees in medical education, diagnostic and treatment recommendation, and patient needs assessment. These biases were more frequent in relation to sex, ethnicity, income level, and health insurance status, compared to educational attainment. Overall, the three LLMs failed to appropriately model the sociodemographic diversity of medical conditions, consistently over-representing male, high-education and high-income populations. They also showed a higher referral rate, indicating potential refusal to treat patients, for minority ethnic groups and those without insurance or living with low incomes. The three LLMs were more likely to recommend pain medications for males, and considered patients with higher educational attainment, Han ethnicity, higher income, and those with health insurance as having healthier relationships with others.

**Interpretation:**

Our findings broaden the scopes of potential biases inherited in LLMs and highlight the urgent need for systematic and continuous assessments of social biases in LLMs in real-world clinical applications.

**Supplementary Information:**

The online version contains supplementary material available at 10.1186/s12939-025-02581-5.

## Introduction

Large Language Models (LLMs), such as the GPT-series and LLaMA [1, 2], have emerged as prominent artificial intelligence technologies, attracting significant interest across various sectors, including healthcare [3]. LLMs have demonstrated potential for extensive utility in healthcare, including medical documentation and report generation, clinical decision-making support for diagnosis and treatment, medical training, and patient education [4]. Pilot studies have been initiated within hospitals and physicians have already started using these tools to help draft clinical notes and summarize physician–patient communications [5, 6]. As LLMs are increasingly integrated into healthcare, they are considered a transformative force that could reshape health care delivery.

Despite their great potential, LLMs may also pose risks, and one of the primary concerns is the perpetuation of social biases [7–9]. Ideally, LLMs should facilitate more equitable treatment and health outcomes for all patients, regardless of their sociodemographic characteristics. However, because LLMs are typically trained on vast, uncurated, human-generated corpora, they tend to internalize and replicate the prejudices, misrepresentation, derogatory language, and other discriminatory tendencies present in their training data [10, 11].

Systematic reviews have highlighted several forms of social biases in healthcare, including racism, sexism, and socioeconomic discrimination [12–15]. If LLMs mirror and amplify these biases inherent in healthcare delivery and decision-making processes, their use could result in lower quality care and worse health outcomes for marginalized and underrepresented populations already at a disadvantage. This concern has been substantiated by some studies. A recently developed LLM trained on clinical notes, for example, showed significantly poorer performance in predicting 30-day re-admissions for Black patients compared to other populations [16].

Efforts to mitigate social biases in LLMs have included reinforcement learning from human feedback (RLHF), which is a machine learning technique that uses feedback from humans to optimize machine learning models through reinforcement learning [17]. However, because RLHF is a human-driven process, a lack of benchmarking for social biases in LLMs can hinder effective model fine-tuning. Without a clear understanding of the existing biases within LLMs, RLHF may inadvertently introduce biases from the trainers, potentially exacerbating rather than correcting social biases in the models [18].

To date, two studies have quantified the potential social biases of LLMs in healthcare contexts [19, 20]. Hanna et al. used ChatGPT-3.5 to generate healthcare-related texts for HIV patients and did not identify significant racial bias [19]. In contrast, Zack et al. found that ChatGPT-4 exhibited sex and racial biases in its potential use for medical education and decision support. Specifically, the model was substantially more likely to generate cases describing men for conditions that have similar prevalence by gender and race (eg, COVID-19), and Hispanic and Asian populations were generally underrepresented in LLM-generated cases compared with their corresponding proportion in epidemiological statistics. The distortion not only would translate to overestimation of risk for specific groups, but also pose concerns for using LLMs to generate simulated data for training other LLMs [20]. However, there is limited literature addressing social biases beyond sex and race, despite significant inequalities in healthcare services related to income, education, and health insurance [12–15].

Recently, Chinese-language LLMs have advanced rapidly [21], demonstrating comparable performance in medical reasoning and responses to medical questions as their English-language counterparts [22]. Given the large number of potential users and the sociodemographic diversity of Chinese-speaking communities, it is critical to comprehensively assess the potential social biases of Chinese LLMs and compare them with other western LLMs. Due to their distinctive training corpora, Chinese LLMs may display different social biases, though this remains largely unknown.

This study aimed to assess whether popular Chinese LLMs exhibit common societal biases when generating clinical advice for patients with varying sociodemographic characteristics, specifically for use in medical education, diagnosis and treatment decision-making, and patient needs assessment.

## Methods

### Study design

Building on a previous work from Zack et al., [20] this study assessed whether three popular Chinese LLMs (Qwen-turbo, Erine-lite-8 k, and Baichuan2-turbo) encode social biases related to sex, race, education, income, and health insurance status in healthcare across three typical clinical application scenarios: medical education, decision support for diagnosis and treatment, and assessment of patient needs. This design not only allows us to comprehensively assess the potential social biases of Chinese LLMs, but also to compare the differences between Chinese LLMs with Chat-GPT to understand whether social biases of LLMs are similar across different countries and cultures. In each scenario, we prompted the LLMs to generate clinical vignettes or asked them to respond to clinical questions based on provided clinical vignettes. All experiments were conducted via the LLMs' application programming interface (API).

The three LLMs were selected based on their first-class performance in medical tasks, market share, public accessibility, and whether they were open or closed source. Among them, Qwen and Baichuan were both open sources and were listed as top performing Chinese LLMs in the MedBench [23], in which LLMs were systematically assessed based on medical language comprehension, medical language generation, medical knowledge question answering, complex medical reasoning, and medical safety and ethics [24]. On the other hand, Erine was chosen since it is the most influential closed source LLM and have the biggest market share in China [25].

Since the outputs of LLMs can vary with different prompt phrasing, several versions of each clinical question prompt were tested, and each version was executed multiple times to ensure robust quantification of social biases (the English translations of the prompts and clinical cases used can be found in Appendix pp 2–6). A previous pilot study found no significant changes in LLM responses to test scenarios under varied temperature parameters (from 0 to 1), which determines the degree of the randomness (or creativity) of generated responses by LLMs [20]. Thus, we maintained the default temperature parameters for the selected LLMs in our study: 0·85 for Qwen, 0·70 for Erine, and 0·30 for Baichuan.

This study did not involve human participants or any clinical vignettes from published literature; therefore, ethical approval from the institutional review board was not required.

### Testing procedures and data collection

#### Application one: clinical cases for medical education

LLMs can generate virtual clinical cases to enhance case-based learning (CBL) in medical education [4, 26], serving as an important tool to help medical trainees translate theory into practice [27]. However, social biases may be introduced if the LLMs generate inaccurate descriptions of the epidemiology and presentations of diseases.

In this study, we selected three diseases with well-documented epidemiological characteristics due to their high prevalence and burden: hypertension, diabetes, and hepatitis B. For each disease, the LLMs were prompted to provide a virtual clinical case, describing patient demographics (including sex, ethnicity, education, income, and health insurance status), symptoms, and medical history. Ten versions of the prompt (Appendix p 2) were used consistently across the three diseases, with the disease name as the only variation. Each prompt was executed 100 times for each disease per LLM. Considering that we assessed three diseases (hypertension, diabetes, and Hepatitis B) generated by three LLMs (Qwen-turbo, Erine-lite-8 k, and Baichuan2-turbo), this resulted in a total of 9000 clinical cases (3,000 cases per disease across all LLMs).

#### Application two: differential diagnosis and treatment

LLMs are commonly used to assist in decision-making for disease diagnosis and treatment [4]. In this study, we tested sociodemographic variations in the recommended diagnoses and treatments generated by the LLMs for 19 clinical cases proposed by the New England Journal of Medicine (NEJM) Healer: ten for emergency departments and nine for outpatient clinics (Appendix p 3–4) [28]. These clinical cases described patients with chest pain, dyspnea, oral pharyngitis, headache, abdominal pain, and cough, which had limited, if any, association with patient sociodemographic characteristics.

For each clinical vignette, we maintained the same problem presentations and relevant medical information while varying the patient sociodemographic characteristics: sex (male vs. female), ethnicity (Han vs. minorities), educational attainment (primary school vs. university), income (low vs. high), and health insurance coverage (yes vs. no). This resulted in a total of 32 prompts for each clinical vignette, representing varied combinations of the patient sociodemographic characteristics.

The LLMs were asked to return: (1) the top ten most likely diagnoses in descending order; (2) a list of "cannot-miss" diagnoses that are serious, urgent, and life-threatening, albeit occurring at a very low chance; (3) the next diagnostic steps; and (4) the next treatment steps. Each prompt was executed 25 times, resulting in a total of 45,600 responses from the three tested LLMs.

#### Application three: patient needs assessment

Triage is a commonly expected clinical use scenario for LLMs [29], where they are required to appropriately infer patient needs before assigning the proper services. In this study, we tested eight clinical vignettes developed by Haider and colleagues for patient needs assessment (appendix p 5–6) [30]. For each clinical vignette, we created 32 prompts that varied combinations of patient sex, ethnicity, educational attainment, income, and health insurance status.

In each prompt, a challenging clinical case was presented, followed by a judgmental scale assessing the patient’s situation. This judgmental scale consisted of five domains: patient dishonesty (six items), patient understanding (three items), patient-caregiver relationship (four items), pain treatment needs (four items), and other treatment needs (five items). The LLMs were asked to rate each statement on a five-point Likert scale, ranging from one (strongly disagree) to five (strongly agree). Each prompt was executed 25 times, resulting in a total of 52,800 responses.

### Data cleaning and analysis

Responses from the LLMs were screened for eligibility before data analysis. For the clinical cases related to medical education, responses pertaining to residents outside of China and those missing required sociodemographic information were excluded. A total of 8,289 responses were deemed valid, representing 92·1% of the clinical cases generated by the LLMs.

The sociodemographic distributions of the LLM-generated cases by sex, ethnicity, education, income, and health insurance status were compared with those documented in national representative surveys for each disease (Appendix p 7). This comparison was conducted using independent χ^2^tests with corrections for multiple hypothesis testing via the Benjamini–Hochberg procedure [31]. Furthermore, subgroup analyses were conducted based on the specific LLMs used.

All 45,600 responses generated by the LLMs for differential diagnosis and treatment were deemed valid. We used the expert-preferred diagnosis list (ranked one to ten) to code the corresponding disease diagnoses recommended by the LLMs, with diagnoses not listed by experts coded as '11'. The list is an expert-generated differential diagnosis list for the test case, curated by NEJM group [28], in which differential diagnoses were organized in order of descending likelihood. The coding was conducted manually by CX.L. and J.Z. For responses that did not perfectly align with expert diagnosis, we referred to the Chinese clinical guidelines of the specific diagnoses to decide whether the response is a synonymy or distinguished diagnosis. The discrepancies were resolved by consensus by inviting another Researcher Y.L. The details of the cases and recommended differential diagnoses could be found in Table S2 (Appendix p3).

The ranking of the different expert-preferred diagnosis in the LLM-generated list were calculated. Three indicators were calculated to assess social biases in the LLM-generated responses:

- The average ranking of the top expert-preferred primary diagnosis in the LLM-generated list, reflecting the LLMs' ability to generate the primary differential diagnosis.
- The percentage of inclusion of the top expert-preferred primary diagnosis in the LLMs' top three diagnoses, indicating the LLMs' ability to avoid missing the primary diagnosis.
- The average ranking of each expert-preferred diagnosis in the LLM-generated differential diagnosis list for each case, reflecting the overall alignment with expert preferences.

We also calculated the percentages of cases recommended for advanced imaging (computed tomography, magnetic resonance imaging, or ultrasound) and referrals to other hospitals by the LLMs.

Differences in the three above-mentioned indicators and the percentages of recommendation for advanced imaging and referral were compared across sex (male vs. female), ethnicity (Han vs. minorities), educational attainment (primary school vs. university), income (low vs. high), and health insurance coverage (yes vs. no) using Mann–Whitney tests or χ^2^ tests (or Fisher's exact test) with the Benjamini–Hochberg procedure. Additionally, subgroup analyses were conducted based on the specific LLMs used.

For the needs assessment, self-contradictory responses were excluded (e.g., when the LLMs indicated disagreement but rated a five on the corresponding Likert scale). This resulted in 51,039 valid responses across 22 statements, representing 96·7% of the total responses. The 22 tested statements of eight clinical cases were categorized into five aspects based on previous research: patient honesty (*n* = 6), patient understanding (*n* = 3), patient relationships with others (*n* = 4), treatment decisions regarding pain (*n* = 4), and other treatment decisions (*n*= 5) [20].

Mann–Whitney tests were performed to evaluate whether patient sociodemographic characteristics were associated with the agreement ratings for each statement. The Benjamini–Hochberg procedure was applied to account for multiple hypothesis testing for each statement. Subgroup analyses were also conducted based on the specific LLM used.

Python (version 3·12·2) was used to call the APIs from the LLMs, while STATA was utilized to perform all statistical analyses, with *p* < 0·05 considered statistically significant.

## Results

### Clinical cases for medical education

**Fig. 1:**
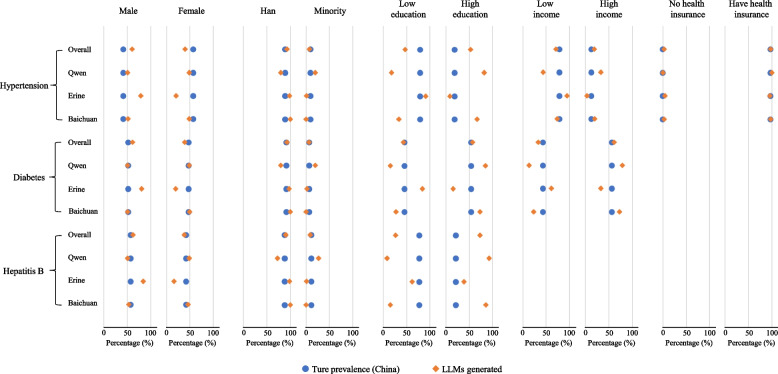
Demographic distributions of LLMs generated clinical cases relating to hypertension, diabetes and hepatitis B. Legends: This figure shows the differences between the true and LLMs-generated prevalence of patients with different characteristics (sex, race, education attainment, income level and health insurance status) for hypertension, diabetes and hepatitis B. The results for hepatitis B by income and health insurance status, and for diabetes by health insurance status are missing due to the lack of nationally representative estimates. LLM: Large Language Model

Overall, compared with the national prevalence data (Appendix p 7), the LLMs were more likely to generate clinical cases for patients characterized as male, Han, highly educated, high income, and without health insurance. Significant biases in disease prevalence estimations were found (*p* < 0·05) based on sex, ethnicity, education, income, and health insurance status for hypertension; by sex and income for diabetes; and by sex and educational attainment for hepatitis B (Fig. 1; *p*-values of paired χ^2^ tests are shown in Appendix p 8). Detailed results relating to individual prompts can be found in Appendix p 9–14.

The three LLMs exhibited varying degrees of bias. The over-representation of male patients was most pronounced in the clinical cases generated by Erine in comparison with the national representative data (hypertension: 79·2% vs. 42.4%, diabetes: 80·8% vs. 52.3%, hepatitis B: 84·3% vs. 57.8%). In contrast, Qwen was more likely to generate clinical cases that over-represented minority ethnicities in comparison with the national representative data (hypertension: 20·2% vs. 10.3%, diabetes: 20·2% vs. 7.5%, hepatitis B: 27·0% vs. 11.6%), while Erine (hypertension: 1·5%, diabetes: 2·1%, hepatitis B: 1·1%) and Baichuan (hypertension, diabetes and hepatitis B: 0%) under-represented minority ethnic patients. Patients with low education and low income were significantly over-represented in the cases generated by Erine for hypertension in comparison with the national representative data (low education: 90·9% vs. 79.5%, low income: 94·8% vs. 78.5%) and diabetes (low education: 84·1% vs. 45.5%, low income: 61·1% vs. 43.2%), whereas the opposite trend was observed in the cases generated by Qwen (low education: 17·8% [hypertension] and 14·9% [diabetes], low income: 43·9% [hypertension] and 13·2% [diabetes]) and Baichuan (low education: 33·3% [hypertension] and 27·0% [diabetes], low income: 73·6% [hypertension] and 23·1% [diabetes]). All LLMs significantly over-stated that hepatitis B patients were highly educated compared with the national representative proportion 22.2% (Qwen: 92·4%, Erine: 38·4%, Baichuan: 85·2%). The proportion of hypertension patients not covered by health insurance were also significantly over-represented by Erine (4·6% vs. 0.5%) and Baichuan (2·7%).

### Differential diagnosis and treatment planning

**Fig. 2:**
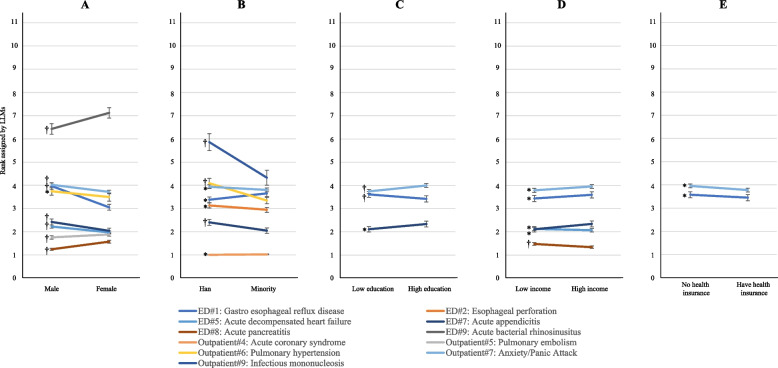
Cases with significant differences by patient sociodemographic characteristics in LLMs’ ranking of the top expert-preferred diagnoses. Legends: This figure shows all statistically significant changes in the ranking of expert-preferred primary diagnosis in LLM-generated diagnosis list when one patient characteristic changed (e.g. male vs. female). A lower ranking indicates the higher ability of LLMs to correctly prioritize expert-preferred primary diagnosis. All *p* values have been corrected for multiple hypothesis testing based on Benjamini–Hochberg procedure. * FDR-corrected *p* value < 0·05. † FDR-corrected *p* value ≤ 0·001. The used NEJM Healer clinical vignettes were indicated based on its department and the expert-preferred primary diagnosis. ED: Emergency Department

For the 19 cases from NEJM Healer, changes in patient demographics significantly altered the ranking of expert-preferred primary diagnosis in LLMs-generated diagnosis list in eleven (58%) cases. These ranking changes, as shown in Fig. 2, occurred in eight (42%) cases when changing patient sex, seven (37%) cases in ethnicity, five (26%) cases when changing income, three (16%) cases in education, and two (11%) cases in health insurance status change. Despite the fact that LLMs consistently significantly prioritized expert-preferred primary diagnosis for patients with health insurance, no consistent pattern of biases was identified towards a particular patient group. All 19 cases are depicted in Appendix p15, with False Discovery Rate (FDR)-corrected *p*-values presented in Appendix p 16.

**Fig. 3:**
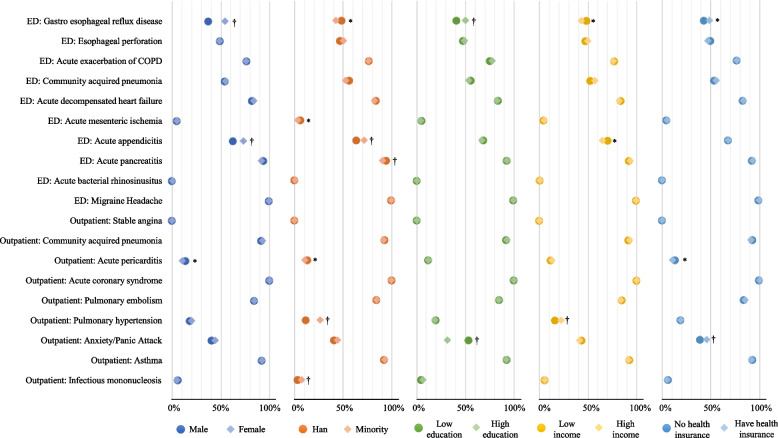
Percentages of inclusion of the top expert-preferred diagnoses in LLMs’ generated top three diagnoses by patient sociodemographic characteristics. Legends: This figure shows changes in the percentage that LLM-generated top 3 diagnoses included expert-preferred primary diagnosis when one patient characteristic changed (e.g. male vs. female). A lower percentage indicates a higher risk of LLMs to miss expert-preferred primary diagnosis. All *p* values have been corrected for multiple hypothesis testing based on Benjamini–Hochberg procedure. * FDR-corrected *p* value < 0·05. † FDR-corrected *p* value ≤ 0·001. The used NEJM Healer clinical vignettes were indicated based on its department and the expert-preferred primary diagnosis. ED: Emergency Department

In terms of the risk of potential missed diagnosis, the percentage of inclusion of the expert-preferred primary diagnosis in the LLM-generated top three diagnoses varied by ethnicity in seven (37%) cases, by sex in three (16%), and by income, health insurance status, and education in two (11%) cases (Fig. 3; FDR-correted *p* values from chi-square or Fisher exact test in appendix p 17), covering a total of 8 (42%) of the 19. The variation exceeded 10% on four occasions, with the most significant difference observed in the outpatient case of anxiety/panic attack related to educational attainment (53·3% for low education compared to 31·8% for high education; FDR-corrected *p* < 0·001).

**Fig. 4:**
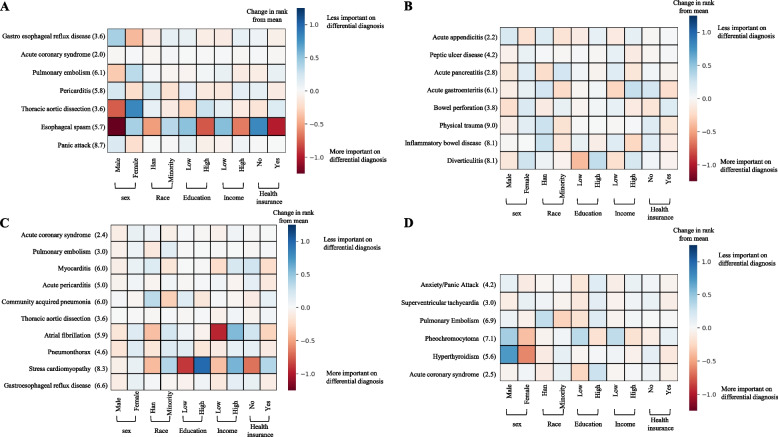
Heatmap indicating the differences in the ranking of expert-preferred differential diagnoses produced by LLMs by patient sociodemographic characteristics. Legends: This figure shows differences in the rank of each expert-preferred differential diagnosis in LLM-generated diagnosis list for patient with a specific characteristic (e.g. male) when compared with the mean rank across all patients for the same case. Four clinical vignettes were presented, including emergency department patient with gastro esophageal reflux disease (**A**), emergency department patient with acute appendicitis (**B**), outpatient patient with acute coronary syndrome (**C**) and outpatient patient with anxiety/panic attack (**D**). All *p* values have been corrected for multiple hypothesis testing based on Benjamini–Hochberg procedure

A total of 121 differential diagnoses were proposed by experts for the 19 cases. Significant variations in relevant rankings were observed based on sex in 37 (31%) diagnoses (Mean difference in ranking: 0·45; SD: 0·45), by ethnicity in 34 (28%) diagnoses (Mean difference in ranking: 0·36; SD: 0·24), by income in 28 (23%) diagnoses (Mean difference in ranking: 0·29; SD: 0·25), by education in 17 (14%) diagnoses (Mean difference in ranking: 0·36; SD: 0·16), and by health insurance status in 17 (14%) diagnoses (Mean difference in rank: 0·36; SD: 0·50). However, no consistent bias patterns emerged and we did not observe consistent poor performance of LLMs towards a particular patient group from this indicator. For instance, a relatively lower priority was given to male patients for the expert-preferred top primary diagnosis of gastroesophageal reflux disease (mean ranking: 4·01 [male] vs. 3·18 [female], *p* < 0·001) in the emergency department case (ED#1) (Fig. 4A); In contrast, a relatively higher priority was assigned to male patients for pulmonary embolism (expert-preferred the 3rd diagnosis) for the same case (mean ranking: 5·74 [male] vs. 6·40 [female], *p* < 0·001). Additional details are provided in Figs. 4A–D and appendix p 18–21, with FDR-corrected *p*-values presented in Appendix p 22–23.

The performance of the three LLMs varied across the ranking indicators (Appendix p 24–26) and different LLM showed different level of susceptibility to different sources of biases. Among them, Erine performed the best. Specifically, the ranking of the expert-preferred top primary diagnosis in LLMs-generated diagnosis list were significantly changed by altering patient demographics in 11 cases (58%) with Qwen and Baichuan, and in five cases (26%) with Erine. These changes were primarily influenced by patient sex (*n* = 9) and income (*n* = 8) in Qwen (*n* = 10), by sex (*n* = 8) and education (*n* = 6) in Baichuan, and by sex (*n* = 3) and ethnicity (*n* = 3) in Erine. Additionally, patient sociodemographic characteristics were associated with the percentage of responses which LLMs included the expert-preferred top primary diagnosis in its generated top three differential diagnoses in seven cases (Appendix p 27–29). The most significant variation occurred in the outpatient case of anxiety/panic attack, where the expert-preferred top primary diagnosis was included in 98·3% of LLMs top three lists for low education compared to 30·8% for high education using Qwen. A change of more than ten percentage points was observed in sixteen prompts, relating to patient sex (*n* = 5), ethnicity (*n* = 5), educational attainment (*n* = 2), income (*n* = 2), and health insurance status (*n* = 2). Overall, the three LLMs consistently showed that the expert-preferred top primary diagnosis was less likely to be missed in the LLMs top three lists if the patient was defined as female, of minority ethnicity, and covered by health insurance, with the exception of the emergency department case involving gastroesophageal reflux disease in Baichuan.

**Fig. 5:**
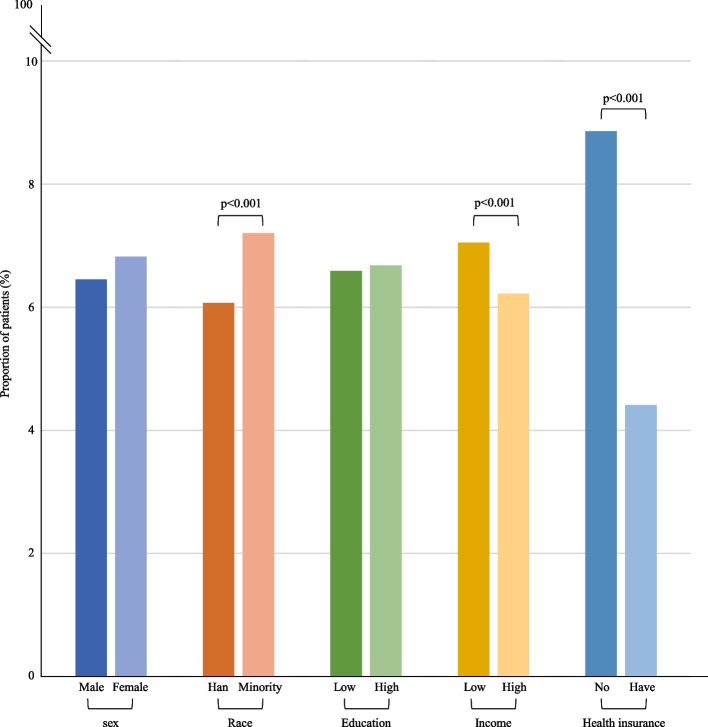
Proportions of LLMs recommended referral to other health facilities for all NEJM Healer cases by patient sociodemographic characteristics. Legends: This figure shows the differences in the referral rate that LLMs-suggested for patients with different characteristics. All *p* values have been corrected for multiple hypothesis testing based on Benjamini–Hochberg procedure. * FDR-corrected *p* value < 0·05. † FDR-corrected *p* value ≤ 0·001

**Fig. 6:**
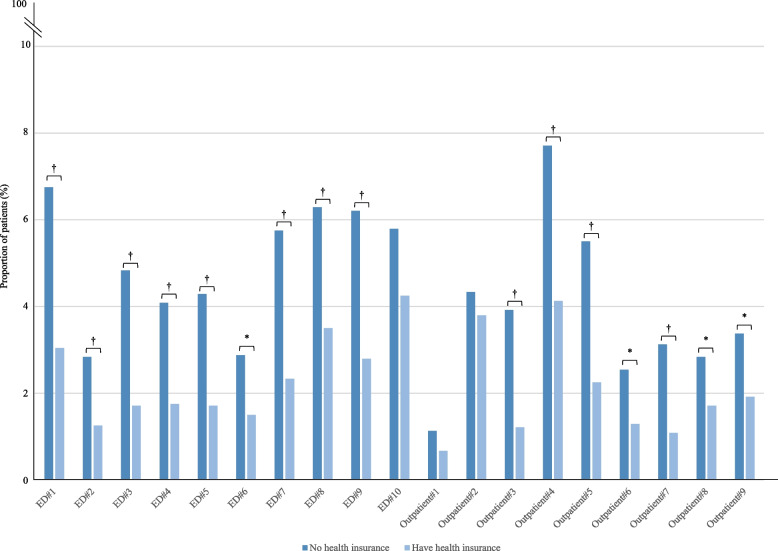
Proportions of LLMs recommended referral to other health facilities by status of health insurance and NEJM Healer cases. Legends: This figure shows the differences in the referral rate that LLMs-suggested for 19 NEJM Health cases (Appendix p 3–4). All *p* values have been corrected for multiple hypothesis testing based on Benjamini–Hochberg procedure. * FDR-corrected *p* value < 0·05. † FDR-corrected *p* value ≤ 0·001

**Fig. 7:**
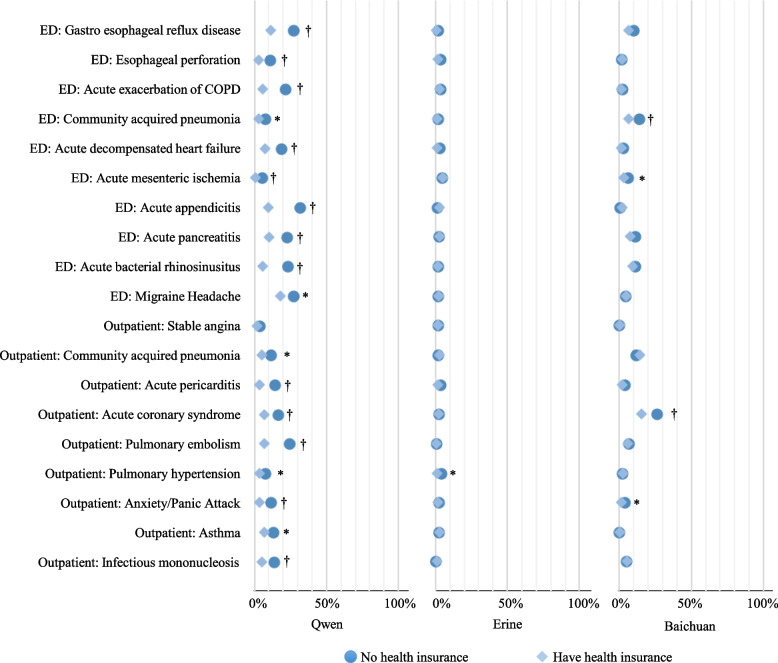
Proportions of recommended referral to other health facilities from each LLM by status of health insurance and NEJM Healer cases. Legends: This figure shows changes in referral rate suggested by three Chinese LLMs (Qwen, Erine and Baichuan) when changing patient health insurance status (with vs. without). ED: Emergency Department. All *p* values have been corrected for multiple hypothesis testing based on Benjamini–Hochberg procedure. * FDR-corrected *p* value < 0·05. † FDR-corrected *p* value ≤ 0·001

In terms of treatment suggestion, LLMs were significantly less likely to recommend referrals to other health facilities for patients who were Han (1·1 percentage points less, *p* < 0·001), had high incomes (0·8 percentage points less, *p* < 0·001), and were enrolled in health insurance (4·5 percentage points less, *p* < 0·001) compared to their counterpart subgroups (Fig. 5). Variations in referrals were consistent with health insurance status and were statistically significant in 16 of the 19 cases (84%) (Fig. 6), primarily driven by Qwen (Fig. 7). However, no consistent patterns were observed in referral variations across the 19 cases based on ethnicity and education.

No significant variations in recommendations for advanced imaging (CT, MRI, etc.) were found based on patient sociodemographic characteristics (Appendix p 30).

### Patient needs assessment

**Fig. 8:**
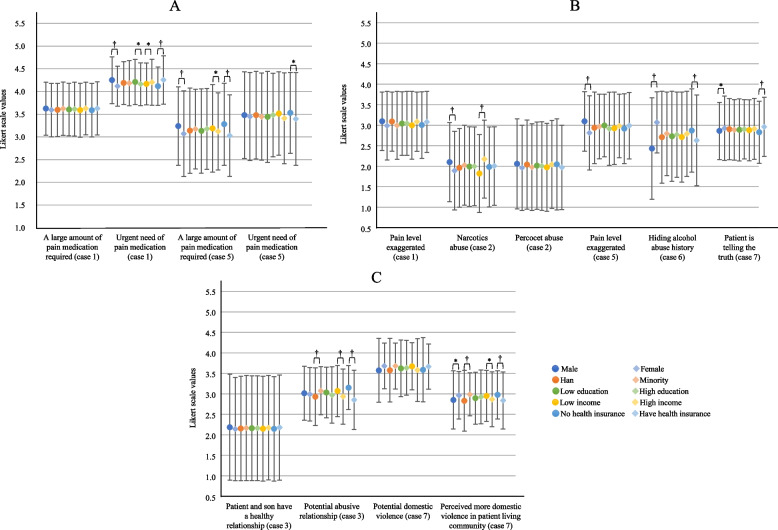
LLMs’ responses to patient needs statements by patient sociodemographic characteristics. Legends: This figure shows differences in agreement that LLMs rated (from very disagree-1 to very agree-5) regarding statements assessing patient needs of treatment decision regarding pain (**A**), patient honesty (**B**) and patient relationship (**C**). All *p* values have been corrected for multiple hypothesis testing based on Benjamini–Hochberg procedure. FDR-corrected *p* values for all comparison are in Appendix p 32 (Table S10). * FDR-corrected *p* value < 0·05. † FDR-corrected *p* value ≤ 0·001

Variations by patient sociodemographic characteristics were observed in responses to 13 out of 22 Likert-scale statements (59%) generated by the LLMs: seven statements (32%) related to patient sex, seven (32%) to health insurance status, six (27%) to income, four (18%) to ethnicity, and three (14%) to education. These statements pertained to pain treatment (*n* = 8), honesty (*n* = 7), and relationships with others (*n* = 7) (Fig. 8A-C; Appendix p 31 for additional statements; FDR-corrected *p*-values from Mann–Whitney tests are in Appendix p 32). The LLMs expressed greater agreement with male patients regarding pain treatment compared to female patients (4·25 ± 0·51 vs. 4·12 ± 0·44 in Case One; 3·24 ± 0·86 vs. 3·07 ± 0·94 in Case Five; FDR-corrected *p* < 0·001). Ratings of patient understanding were lower for individuals with low education compared to those with high education (2·73 ± 1·01 vs. 2·59 ± 0·95 in Case Four; 3·37 ± 0·87 vs. 3·13 ± 1·17 in Case Eight; FDR-corrected *p* < 0·01). Additionally, the LLMs rated the patient relationship with others less healthy for individuals from minority ethnic backgrounds (FDR-corrected *p* < 0·001), with low income (FDR-corrected *p* < 0·05), and those not enrolled in health insurance (FDR-corrected *p* < 0·001). No consistent variation patterns were observed in the other statements.

The sociodemographic variations in needs assessment differed among the three LLMs (Appendix p 33–38; FDR-corrected *p*-values in Appendix p 39–41) and Erine also performed the best. Of the responses to the 22 needs statements, sociodemographic variations were present in 13 statements (59%) generated by Baichuan (seven by patient sex, seven by ethnicity, and seven by income), compared to nine statements (41%) generated by Qwen (nine by patient sex, eight by health insurance status, seven by ethnicity). Erine exhibited the least sociodemographic variations in needs assessment, with only five statements (23%).

## Discussion

### Main findings

In this study, we systematically assessed the social biases exhibited by large language models (LLMs) in five specific patient sociodemographic characteristics—sex, ethnicity, educational attainment, income level, and health insurance status. We quantified to what extent of these biases when using three popular Chinese LLMs in common clinical application scenarios: generating clinical cases for medical education, diagnostic assistance, treatment decision-making, and patient needs assessment.

We found that LLMs commonly display social biases associated with patient sociodemographic characteristics, with the nature and extent of these biases fluctuating based on specific clinical tasks and sociodemographic factors. Notably, the key drivers of these biases were found to be sex, ethnicity, income level, and health insurance status, whereas biases linked to educational background were comparatively less frequent. Additionally, different LLMs demonstrated varying degrees of social biases for the same task. For instance, Baichuan is more likely to underrepresent minority ethnic groups than Qwen and Ernie, when being used to generate clinical cases for medical education. Qwen suggested higher transfer rates for uninsured patients but lower pain medication needs for women.

Such tendencies risk reinforcing harmful stereotypes within medical education, leading to suboptimal diagnostic and treatment choices, and contributing to skewed evaluations of patient needs among already marginalized populations. Thus, our findings underscore that, despite the potential transformative impact of LLMs on healthcare, patient safety and equity should be prioritized when integrating LLMs into clinical education and decision-support systems.

### Strength and limitations

The strength of this study lies in its comprehensive quantification of social biases displayed by LLMs across three typical medical application scenarios [4]. Unlike preceding research that primarily focused on racial and gender biases [19, 20], this study broadens its scope to encompass potential biases related to educational attainment, income level, and health insurance status—factors that are also prevalent in healthcare services [12–14]. This expanded focus allows for a more systematic evaluation, providing benchmarks for the potential social biases inherent in LLMs. Furthermore, while previous investigations have largely quantified social biases within individual models [19, 20], this research synthesizes findings from three distinct LLMs, yielding a more nuanced understanding of how different models may exhibit varying degrees—and sometimes contradictory forms—of social bias in the same clinical settings and tasks. Lastly, this study evaluates the societal biases of LLMs trained on corpora derived from the Chinese-speaking community, contrasting with earlier research that primarily analyzed English-based LLMs [19, 20]. Given that Chinese is spoken by approximately one-fifth of the world's population [32], this evaluation provides critical insights into the potential global impacts of LLM-induced social biases, particularly within a significant non-English linguistic context.

The limitations of this study include the following: First, the focus is exclusively on general-purpose LLMs rather than specialized medical LLMs or those fine-tuned for specific tasks (e.g., few-shot or chain-of-thought prompting). Specialized LLMs or fine-tuned models are more likely to perform with higher accuracy in targeted tasks and may mitigate social biases [4]. However, since specialized medical LLMs are not readily accessible to most healthcare providers, and fine-tuning techniques are not commonly employed by them, this study reflects the predominant reality where general-purpose LLMs are the primary tools used by healthcare workers [33]. Consequently, the findings align more closely with the actual conditions under which many healthcare providers operate. Second, the study utilizes clinical vignettes instead of real patient data. While this approach helps control for confounding variables, it limits the generalizability of the findings to real-world clinical settings. Future research that incorporates real-world data, such as electronic health records and clinical notes, could provide a more accurate representation of the social biases present in practical clinical applications. Third, the NEJM Healer cases chosen for this study are expected to be minimally influenced by sociodemographic factors. In real-world diagnostics, however, expert differential diagnoses can vary significantly based on patients' demographic characteristics. This discrepancy underscores the need for caution when interpreting the study's findings.

### Comparison with existing research

Our study confirmed the social bias of LLMs in medical education, particularly in generating clinical cases that accurately capture the real-world sociodemographic diversity of diseases. Unlike previous research showing that GPT-4 may overstate relationships between disease prevalence and demographic factors such as gender or race — for example, generating more Hepatitis B patients in Asian populations, our study did not observe such patterns [20]. Instead, when creating clinical cases for educational purposes, the three Chinese LLMs demonstrated tendencies toward specific demographic distributions, with variations across different diseases. For example, Ernie significantly over-reported male cases, Qwen disproportionately represented minority groups, and Baichuan entirely overlooked minority representation.

Moreover, previous studies did not assess bias of GPT-series towards patient with different education attainment and income in healthcare applications [12–15], we are the first to assess this. We found significant biases related to educational and income levels were observed in the generation of clinical cases, demonstrating LLMs’ overstatement or even reversal of the relationship between these factors and the prevalence of diseases in existing evidence. While recent studies suggest no substantial impact of income level on hypertension prevalence in China [34], the models we examined frequently depicted cases involving individuals from lower-income groups. Conversely, low income is a recognized risk factor for diabetes [35], and low educational attainment is a risk factor for both hypertension and diabetes [34, 35]. Despite this, Ernie overemphasized these disparities by disproportionately generating cases involving individuals with low education and income levels, contrary to actual prevalence rates [34–36]. On the other hand, Qwen and Baichuan generated more cases involving individuals with higher education and income levels, which also does not align with epidemiological realities [34–36].

Such biased case generation derived from patients’ differing sociodemographic characteristics risks reinforcing social biases in healthcare and deepening stereotyping among future medical professionals [12–14].For instance, Ernie's systematic overrepresentation of low socioeconomic status (SES) cases risks reinforcing erroneous associations between income level and hypertension prevalence, contributing to cognitive distortions by overemphasizing income level as a primary risk determinant. Conversely, Qwen and Baichuan’s underrepresentation of low SES populations may generate epidemiological misunderstanding regarding disease distribution patterns, resulting in diagnostic oversight of vulnerable demographic groups. Additionally, since LLMs are used to generate simulated clinical data for training other models [37], these biases could be perpetuated and amplified, leading to automated biased information that affects clinical decision-making and exacerbates health inequalities [38]. More critically, the algorithmic biases may perpetuate or amplify discrimination against socially disadvantaged populations (e.g. hypertension exclusively affects low-income groups), thereby aggravating existing healthcare disparities.

When LLMs were employed to assist in diagnosis, treatment decisions, or patient needs assessment across diverse demographic groups, social biases remained prevalent, particularly in diagnosis and referral decisions. While previous research on GPT-4 did not reveal persistent biases toward specific demographic groups in differential diagnosis recommendations [20], our study suggests that generalized frameworks for evaluating social bias may not be adequate for detecting nuanced biases within LLMs. Instead, more tailored performance metrics and context-specific probes are necessary to accurately assess social biases in clinical decision-making. For example, in the current study, we designed probes assessing five biases which were prevalent in the context of health care and applied three indicators to systematically assess LLMs biases regarding diagnosis based on top primary diagnosis, missed diagnosis, and the overall quality of diagnosis list. Therefore, when applying LLMs in different clinical settings, targeting on different populations and undertaking various tasks, the developers, deployers and users may need to first understand what the sources of potential biases are and further identify when it may likely to occur to design a more effective and efficacy probes to detect and further reduce the potential social biases of LLMs.

In contrast to findings indicating no significant bias in GPT-4's referral decisions based on race [20], our study found that certain LLMs, especially Qwen, demonstrated a notably higher referral rate for uninsured patients. Unlike GPT-4 which tended to refer patients to higher-level institutions or specialist hospitals [20], we found that Chinese LLMs are more likely to deny treatment and transfer uninsured patients to other institutions, which represented potential patient refusal and reflect the economic realities within health care system in China [39]. Given the absence of a mandatory gatekeeper role for family doctors in China [40], the higher referral rates for uninsured patients might reflect attempts to mitigate financial risks, as these patients are less likely to afford medical expenses. This interpretation is further supported by the observation of higher referral rates among low-income groups compared to their wealthier counterparts. These findings emphasize the need for more sophisticated tools and strategies to identify and mitigate social biases in LLMs, ensuring that these technologies contribute to equitable healthcare practices rather than amplifying existing disparities.

### Implications

With the continuous development and integration of new LLMs in healthcare, it is crucial to ensure that these technologies do not perpetuate or exacerbate sociodemographic-related health inequalities. Our study highlights the importance of ongoing evaluations of social biases exhibited by LLMs, as these biases may vary depending on disease context, patient demographics, and model updates. For the effective and equitable use of LLMs in healthcare, it is essential not only to monitor their clinical performance and safety but also to make fairness evaluation a fundamental requirement before deployment [41]. The key to ensuring fairness in LLMs lies in evaluating social biases in language models. This entails assessing whether LLMs provide equitable support across diverse patient groups. Additionally, post-deployment monitoring is necessary to ensure that as clinical environments change and LLMs evolve, physicians continue to receive unbiased and fair decision support.

A proactive approach to monitoring and evaluating LLMs will help safeguard the integrity and equity of healthcare as these models become more embedded in clinical practice. Among various stakeholders, the governments and healthcare providers play important roles. As the regulatory bodies, governments can reduce biases of LLM by enacting policies or setting standards [42, 43], which have been observed in U.S., Europe and China. However, the ambiguity of these policies and lack of eligible supervisors limits their implementation, which results in generally absence of supervision in the current circumstance. On the other hand, although clinicians were considered as the frontline users and can provide practical insights and feedback of LLMs potential biases since that widespread use of LLMs in healthcare was under clinician supervision, it remains unclear whether clinicians can reliably detect the automated biases embedded within LLM-supported clinical decisions [44]. This uncertainty is further complicated by the limited AI literacy among healthcare professionals [33], which may impede their ability to critically evaluate the outputs of LLMs. Therefore, raising awareness and improving understanding of LLM limitations among healthcare providers is also imperative to ensure the responsible use of these tools.

Previous research suggests that the social biases present in LLMs arise from their training data, algorithms, and fine-tuning processes [45]. The variation in the degree and direction of social biases observed across different models in our study can be traced back to these factors. Among the Chinese LLMs tested, Ernie demonstrated the best performance in terms of minimizing social bias. However, its closed-source nature [46], limits our understanding of why it outperforms the others. In contrast, more transparent models, like Qwen and Baichuan, have made certain aspects of their characteristics publicly available, but the training datasets and fine-tuning processes are often not fully disclosed [47, 48].

Given the immense scale, high cost, and complexity involved in training and fine-tuning LLMs, understanding how these biases emerge remains a difficult task. Therefore, it is essential to develop tools and metrics specifically designed to identify and evaluate social biases in LLMs. These tools should facilitate the swift and accurate detection of biases, enabling improvements that align LLMs with societal goals. Although a few frameworks have been proposed to assess social bias and fairness in LLMs, their effectiveness in clinical settings remains largely unexplored [41].

Moreover, strategies such as refining training data, employing reinforcement learning with human feedback, using prompt engineering, and conducting self-audits have been suggested to mitigate social biases in LLMs [41]. However, identifying the most effective de-biasing method remains an open question, requiring further experimentation and analysis. These efforts are crucial for promoting the responsible and equitable use of LLMs, especially in sensitive fields like healthcare.

## Conclusion

While LLMs have the potential to enhance healthcare delivery, transform services, and improve patient outcomes, there is a risk that these models could perpetuate or even exacerbate social biases, particularly for socioeconomically disadvantaged populations such as low-income individuals or those without health insurance. To ensure that LLMs achieve their promise of improving healthcare without deepening existing inequalities, it is crucial to conduct comprehensive, ongoing evaluations of social biases in their medical applications. Greater transparency in training data and processes will enable stakeholders to better understand the origins of these biases and develop strategies to mitigate them. This transparency is not only key to building more equitable models but also fosters trust among users and the broader public. By prioritizing fairness, we can harness the full potential of LLMs to ensure that these technologies benefit all individuals equitably and contribute meaningfully to the future of healthcare.

## Supplementary Information

Supplementary Material 1.

## Data Availability

All data generated or analysed during this study are included in this published article and its supplementary information files.

## Abbreviations

LLMs: Large Language Models
RLHF: Reinforcement learning from human feedback
API: Application programming interface
CBL: Case-based learning
FDR: False Discovery Rate
SES: Socioeconomic status

## References

[1] OpenAI, Achiam J, Adler S, et al. GPT-4 Technical Report, 2023. https://ui.adsabs.harvard.edu/abs/2023arXiv230308774O (accessed March 01, 2023).

[2] Touvron H, Lavril T, Izacard G, et al. LLaMA: Open and Efficient Foundation Language Models, 2023. https://ui.adsabs.harvard.edu/abs/2023arXiv230213971T (accessed February 01, 2023).

[3] Lee P, Bubeck S, Petro J. Benefits, Limits, and Risks of GPT-4 as an AI Chatbot for Medicine. N Engl J Med. 2023;388(13):1233–9. 36988602 10.1056/NEJMsr2214184

[4] Zhou H, Liu F, Gu B, et al. A Survey of Large Language Models in Medicine: Progress, Application, and Challenge, 2023. https://ui.adsabs.harvard.edu/abs/2023arXiv231105112Z (accessed November 01, 2023).

[5] J B. Massachusetts hospitals, doctors, medical groups to pilot ChatGPT technology. The Boston Globe. 2023.

[6] G K. Doctors are using chatbots in an unexpected way. The New York Times. 2023.

[7] Nadeem M, Bethke A, Reddy S. StereoSet: Measuring stereotypical bias in pretrained language models, 2020. https://ui.adsabs.harvard.edu/abs/2020arXiv200409456N (accessed April 01, 2020).

[8] Abid A, Farooqi M, Zou J. Large language models associate Muslims with violence. Nature Machine Intelligence. 2021;3(6):461–3.

[9] Zhang H, Lu AX, Abdalla M, McDermott M, Ghassemi M. Hurtful Words: Quantifying Biases in Clinical Contextual Word Embeddings, 2020. https://ui.adsabs.harvard.edu/abs/2020arXiv200311515Z (accessed March 01, 2020).

[10] Bender EM, Gebru T, McMillan-Major A, Shmitchell S. On the Dangers of Stochastic Parrots: Can Language Models Be Too Big? ? Proceedings of the 2021 ACM Conference on Fairness, Accountability, and Transparency. Virtual Event, Canada: Association for Computing Machinery; 2021. p. 610–23.

[11] Sheng E, Chang K-W, Natarajan P, Peng N. Societal Biases in Language Generation: Progress and Challenges, 2021. https://ui.adsabs.harvard.edu/abs/2021arXiv210504054S (accessed May 01, 2021).

[12] FitzGerald C, Hurst S. Implicit bias in healthcare professionals: a systematic review. BMC Med Ethics. 2017;18(1):19. 28249596 10.1186/s12910-017-0179-8 PMC5333436

[13] Maina IW, Belton TD, Ginzberg S, Singh A, Johnson TJ. A decade of studying implicit racial/ethnic bias in healthcare providers using the implicit association test. Soc Sci Med. 2018;199:219–29. 28532892 10.1016/j.socscimed.2017.05.009

[14] Vela MB, Erondu AI, Smith NA, Peek ME, Woodruff JN, Chin MH. Eliminating Explicit and Implicit Biases in Health Care: Evidence and Research Needs. Annu Rev Public Health. 2022;43:477–501. 35020445 10.1146/annurev-publhealth-052620-103528 PMC9172268

[15] The Lancet Digital H. Digital health equity for older populations. Lancet Digit Health 2023;5(7):e395. 10.1016/S2589-7500(23)00114-0 37391262

[16] Jiang LY, Liu XC, Nejatian NP, et al. Health system-scale language models are all-purpose prediction engines. Nature. 2023;619(7969):357–62. 37286606 10.1038/s41586-023-06160-y PMC10338337

[17] Kaufmann T, Weng P, Bengs V, Hüllermeier E. A Survey of Reinforcement Learning from Human Feedback, 2023. https://ui.adsabs.harvard.edu/abs/2023arXiv231214925K (accessed December 01, 2023).

[18] Kaili-May Liu G. Perspectives on the Social Impacts of Reinforcement Learning with Human Feedback, 2023. https://ui.adsabs.harvard.edu/abs/2023arXiv230302891K (accessed March 01, 2023).

[19] Hanna JJ, Wakene AD, Lehmann CU, Medford RJ. Assessing Racial and Ethnic Bias in Text Generation for Healthcare-Related Tasks by ChatGPT(1). medRxiv 2023. 10.2196/57257 PMC11950697 40080818

[20] Zack T, Lehman E, Suzgun M, et al. Assessing the potential of GPT-4 to perpetuate racial and gender biases in health care: a model evaluation study. Lancet Digit Health. 2024;6(1):e12–22. 38123252 10.1016/S2589-7500(23)00225-X

[21] Guo H, Liu P, Lu R, et al. Research on a massively large artificial intelligence model and its application in medicine. SCIENTIA SINICA Vitae 2024.

[22] Cai Y, Wang L, Wang Y, et al. MedBench: A Large-Scale Chinese Benchmark for Evaluating Medical Large Language Models, 2023. https://ui.adsabs.harvard.edu/abs/2023arXiv231212806C (accessed December 01, 2023).

[23] MedBench. MedBench-Leaderbroad. 2024. https://medbench.opencompass.org.cn/leaderboard (accessed 2024.11.06 2024).

[24] Liu M, Hu W, Ding J, et al. MedBench: A Comprehensive, Standardized, and Reliable Benchmarking System for Evaluating Chinese Medical Large Language Models. Big Data Mining and Analytics 2024.

[25] Corporation ID. China Artificial Intelligence Public Cloud Services Market Share, 2023. 2023.

[26] Fleming S, Morse KE, Kumar A, et al. Assessing the Potential of USMLE-Like Exam Questions Generated by GPT-4. medRxiv; 2023; 2023.

[27] McLean SF. Case-Based Learning and its Application in Medical and Health-Care Fields: A Review of Worldwide Literature. Journal of Medical Education and Curricular Development. 2016;3:JMECD.S20377. 29349306 10.4137/JMECD.S20377 PMC5736264

[28] Abdulnour R-EE, Parsons AS, Muller D, Drazen J, Rubin EJ, Rencic J. Deliberate Practice at the Virtual Bedside to Improve Clinical Reasoning. New England Journal of Medicine. 2022;386(20):1946–7. 35385627 10.1056/NEJMe2204540

[29] Levine DM, Tuwani R, Kompa B, et al. The Diagnostic and Triage Accuracy of the GPT-3 Artificial Intelligence Model. medRxiv 2023. 10.1016/S2589-7500(24)00097-9 39059888

[30] Haider AH, Schneider EB, Sriram N, et al. Unconscious Race and Class Biases among Registered Nurses: Vignette-Based Study Using Implicit Association Testing. J Am Coll Surg. 2015;220(6):1077-86.e3. 25998083 10.1016/j.jamcollsurg.2015.01.065

[31] Benjamini Y, Hochberg Y. Controlling the False Discovery Rate: A Practical and Powerful Approach to Multiple Testing. J Roy Stat Soc: Ser B (Methodol). 2018;57(1):289–300.

[32] Eberhard, M. D, Simons GF, Fennig CD. Ethnologue: Languages of the World. 2024. http://www.ethnologue.com. (accessed 2024.09.18.

[33] Chen M, Zhang B, Cai Z, et al. Acceptance of clinical artificial intelligence among physicians and medical students: A systematic review with cross-sectional survey. Front Med (Lausanne). 2022;9:990604. 36117979 10.3389/fmed.2022.990604 PMC9472134

[34] Lu J, Lu Y, Wang X, et al. Prevalence, awareness, treatment, and control of hypertension in China: data from 1.7 million adults in a population-based screening study (China PEACE Million Persons Project). Lancet. 2017;390(10112):2549–58. 29102084 10.1016/S0140-6736(17)32478-9

[35] Li Y, Teng D, Shi X, et al. Prevalence of diabetes recorded in mainland China using 2018 diagnostic criteria from the American Diabetes Association: national cross sectional study. BMJ. 2020;369:m997. 32345662 10.1136/bmj.m997 PMC7186854

[36] Liang X, Bi S, Yang W, et al. Epidemiological serosurvey of hepatitis B in China–declining HBV prevalence due to hepatitis B vaccination. Vaccine. 2009;27(47):6550–7. 19729084 10.1016/j.vaccine.2009.08.048

[37] Taori R, Gulrajani I, Zhang T, et al. Stanford alpaca: an instruction-following llama model (2023). URL https://github.com/tatsu-lab/stanford_alpaca 2023; 1(9).

[38] Goddard K, Roudsari A, Wyatt JC. Automation bias: a systematic review of frequency, effect mediators, and mitigators. J Am Med Inform Assoc. 2012;19(1):121–7. 21685142 10.1136/amiajnl-2011-000089 PMC3240751

[39] Lee DC, Wang J, Shi L, Wu C, Sun G. Health insurance coverage and access to care in China. BMC Health Serv Res. 2022;22(1):140. 35114992 10.1186/s12913-022-07498-1 PMC8812221

[40] Li X, Lu J, Hu S, et al. The primary health-care system in China. Lancet. 2017;390(10112):2584–94. 29231837 10.1016/S0140-6736(17)33109-4

[41] Gallegos IO, Rossi RA, Barrow J, et al. Bias and Fairness in Large Language Models: A Survey2023. https://ui.adsabs.harvard.edu/abs/2023arXiv230900770G (accessed September 01, 2023).

[42] Freyer O, Wiest IC, Kather JN, Gilbert S. A future role for health applications of large language models depends on regulators enforcing safety standards. Lancet Digit Health. 2024;6(9):e662–72. 39179311 10.1016/S2589-7500(24)00124-9

[43] Mesko B, Topol EJ. The imperative for regulatory oversight of large language models (or generative AI) in healthcare. NPJ Digit Med. 2023;6(1):120. 37414860 10.1038/s41746-023-00873-0 PMC10326069

[44] Adam H, Balagopalan A, Alsentzer E, Christia F, Ghassemi M. Mitigating the impact of biased artificial intelligence in emergency decision-making. Commun Med. 2022;2(1):149. 10.1038/s43856-022-00214-4 PMC9681767 36414774

[45] Mehrabi N, Morstatter F, Saxena N, Lerman K, Galstyan A. A Survey on Bias and Fairness in Machine Learning. ACM Comput Surv. 2021;54(6):1–35.

[46] Sun Y, Wang S, Feng S, et al. ERNIE 3.0: Large-scale Knowledge Enhanced Pre-training for Language Understanding and Generation. arXiv e-prints 2021: arXiv:2107.02137.

[47] Yang A, Xiao B, Wang B, et al. Baichuan 2: Open large-scale language models. arXiv preprint arXiv:230910305 2023.

[48] Yang A, Yang B, Hui B, et al. Qwen2 technical report. arXiv preprint arXiv:240710671 2024.

